# Refractory Thrombocytopenia Responds to Octreotide Treatment in a Case of Evans Syndrome with Gastric Neuroendocrine Tumor

**DOI:** 10.1155/2013/391086

**Published:** 2013-07-31

**Authors:** Kocfa Chung-Delgado, Alejandro Revilla-Montag, Sonia Guillén-Bravo, Hugo Ríos-Díaz, José C. Alva-Muñoz

**Affiliations:** ^1^Escuela de Medicina, Universidad Peruana de Ciencias Aplicadas, Lima 33, Peru; ^2^Hematology Department, Hospital Nacional “Edgardo Rebagliati Martins”, Lima 33, Peru; ^3^Clinical Pathology Department, Hospital Nacional “Edgardo Rebagliati Martins”, Lima 33, Peru

## Abstract

A 37-year-old woman with history of Evans Syndrome with poor response to high-dose corticoid treatment presented to the emergency department with gastrointestinal and vaginal bleeding. The patient was later diagnosed with severe thrombocytopenia and a stage G1, well-differentiated gastric neuroendocrine tumor, confirmed by a biopsy. A total gastrectomy was performed to eradicate the tumor. After being treated with a total splenectomy for her Evans Syndrome with no clinical or laboratory improvement, she began regular treatment with octreotide on the basis of a possible hepatic metastasis. Days after the initiation of the octreotide, an increase in the platelet count was evidenced by laboratory findings, from 2,000 platelets/mm^3^ to 109,000 platelets/mm^3^. Weeks later, the hepatic metastasis is discarded by a negative octreotide-body scan, and the octreotide treatment was interrupted. Immediately after the drug interruption, a progressive and evident descent in the platelet count was evidenced (4000 platelets/mm^3^). The present case report highlights the possible association between octreotide treatment and a severe thrombocytopenia resistant to conventional treatment.

## 1. Background

Evans Syndrome is a condition involving the simultaneous or sequential presence of the following conditions: autoimmune hemolytic anemia and idiopathic thrombocytopenic purpura. In both cases, the red blood cells or the platelets are matched with autoantibodies to begin their process of opsonization and their evident premature destruction by the reticuloendothelial system. Evans Syndrome is a chronic condition which is characterized by the presence of flares and exacerbations [1]. Many treatment options are available for this condition; however, in many cases, the disease might require splenectomy as a second-line treatment [2].

On the other hand, a carcinoid tumor is a rare type of neoplasm derived from enterochromaffin-like cells. Because of intrinsic properties, these tumors have the ability of secreting high quantities of hormones such as serotonin, histamine, gastrin, and prostaglandins, among others; serotonin-secreting cells being the most common amongst them [3]. The clinical presentation of the carcinoid-derived tumors has been associated with multiple paraneoplastic syndromes [4, 5]. 

The following case report presents a patient with history of Evans Syndrome with poor response to high-dose corticoid treatment, associated with the presence of a gastric neuroendocrine carcinoid tumor, which has evolved favorably with the use of octreotide. 

## 2. Case Presentation

A 37-year-old woman with history of inactive Evans Syndrome with poor response to high-dose corticoid treatment presented to the emergency department with sudden hematemesis, persistent vaginal bleeding and severe thrombocytopenia. A gastric biopsy taken by upper endoscopy revealed a stage G1 well-differentiated infiltrative neuroendocrine tumor (NET), compatible with a carcinoid tumor. Other possible diagnoses such as chronic or atypical infections, rheumatoid diseases and other bone marrow pathologies were discarded (Table 1). 

After unsuccessful treatment with vincristine, danazol, cyclophosphamide and cyclosporine, the patient underwent a total splenectomy as a second-line treatment to her Evans Syndrome which had a last platelet count of 5,000 platelets/mm^3^. The splenectomy had a poor response. The pathology of the spleen showed moderate congestion of the red pulp without any additional alterations. 

**Figure 1:**
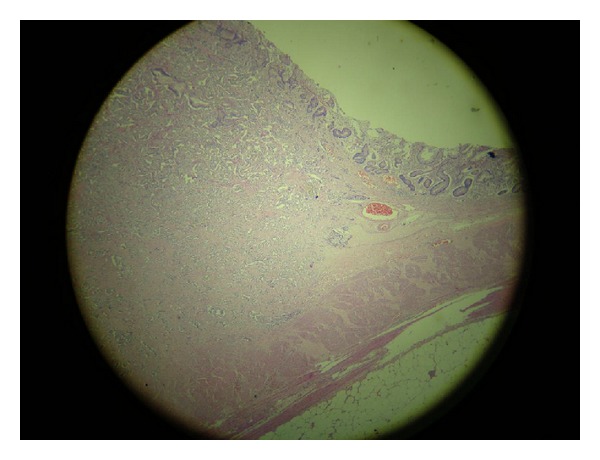
Carcinoid neuroendocrine tumor, stage 1, with infiltration to the subserosal layer, associated with autoimmune atrophic gastritis.

**Figure 2:**
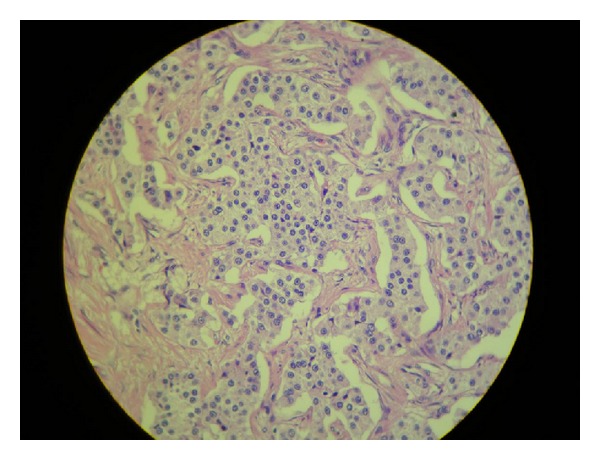
Carcinoid neuroendocrine tumor, stage 1, with infiltration to the subserosal layer, associated with autoimmune atrophic gastritis.

**Figure 3:**
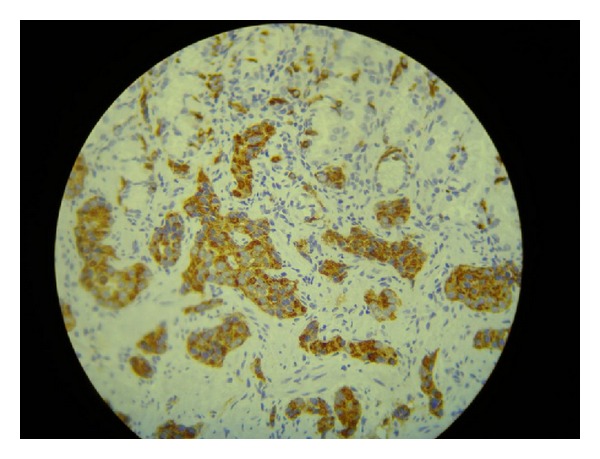
Immunohistochemistry exam positive for chromogranin A.

**Figure 4:**
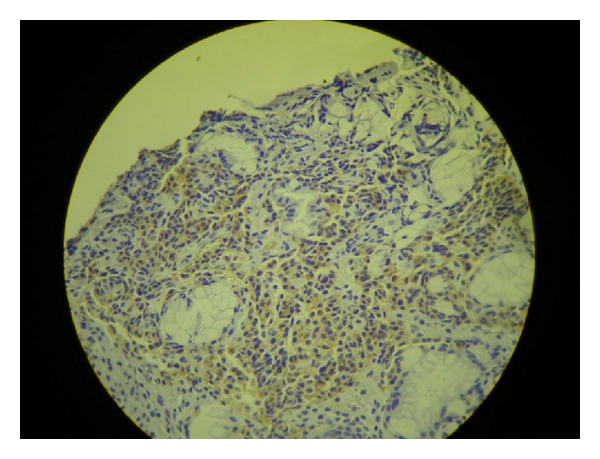
Immunohistochemistry exam positive for synaptophysin.

**Figure 5:**
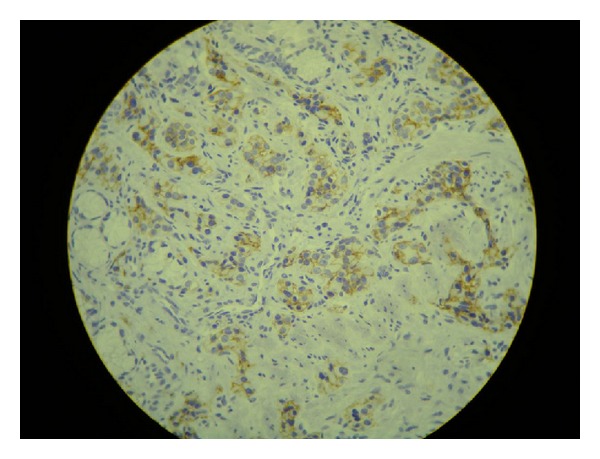
Immunohistochemistry exam positive for CD56.

Seven weeks later, the patient went through a total gastrectomy as a definite treatment for her NET after repeated episodes of hematemesis. Her presurgical exams showed the following results: gastrin = 927 ng/L (1–100 ng/L); 5-hydroxyindoleacetic acid 5-HIAA = 2.1 mg/24 h (0–10 mg/24 h); chromogranin A = 142 ng/mL (1.9–15 ng/mL). The pathology of the surgical specimen confirmed the initial diagnosis: carcinoid NET, stage 1, with infiltration to the subserosal layer, associated with atrophic gastritis (Figures 1 and 2). The immunohistochemistry exam tested positive for chromogranin A (Figure 3), positive for synaptophysin (Figure 4), positive for CD56 marker, (Figure 5), positive for CD8 marker and negative for CD4 marker. 

The patient was prescribed high-dose corticoids, immunoglobulin G, and rituximab but remained hospitalized due to persistent severe thrombocytopenia. Her postsurgical exam values were: gastrin = 26 ng/L; chromogranin A = 3.2 ng/mL. 

**Figure 6:**
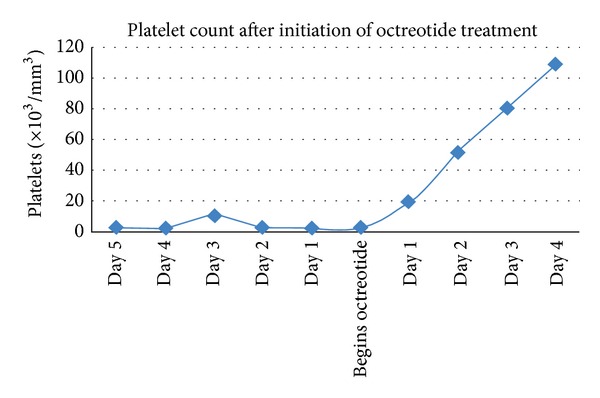
Platelet count after initiation of octreotide treatment.

Two months later, the patient was reevaluated due to acute abdominal pain. Nodular hepatic lesions and severe thrombocytopenia (2,000 platelets/mm^3^) were found during the workup. Due to the high suspicion of hepatic metastasis of the NET, she was started on octreotide, 50 *μ*g/8 h subcutaneous (SQ). Immediately after the first doses of octreotide, a constant increase in the platelet count was evidenced in the CBC, reaching 109,000 platelets/mm^3^ (Figure 6). She was discharged with the same dosage of octreotide. 

Over the following months, the patient showed clinical improvement. She was then changed to octreotide LAR depot medication, 40 mg every 30 days. Consequently, over the following 4 weeks, the platelet count decreased to 2,400 platelets/mm^3^. She was readmitted into the emergency department with petechiae on her thorax and limbs. Conventional octreotide at 50 *μ*g/8 h SQ was started until the possible hepatic metastasis was discarded. An elevation of platelet count to 164,000 platelets/mm^3^ was seen. A complete set of exams were executed to rule out any residual activity of the NET: chromogranin A = 4.8 ng/mL; 5-HIAA = 2.4 mg/24 h; octreotide-Tc99 full-body scan negative, PET-SCAN negative for somatostatin-secreting lesions. With the lab and image results, the Endocrinology Department discarded the possibility of active NET, consequently discontinuing the administration of octreotide. 

**Figure 7:**
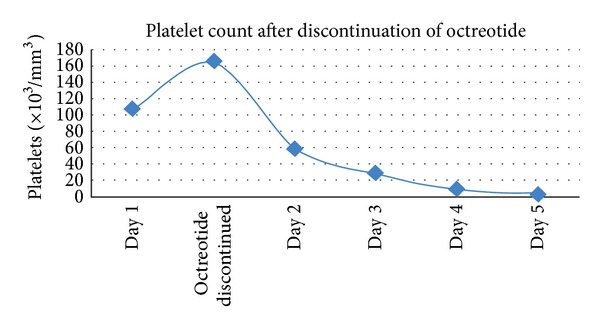
Platelet count after discontinuation of octreotide.

An abrupt fall in the platelet count was noted over the following 5 days (4,000 platelets/mm^3^) after the discontinuation of octreotide (Figure 7). 

## 3. Discussion and Conclusions

We report the case of a 37-year-old woman with Evans Syndrome which responded poorly to conventional first-line treatment and an infiltrative gastric neuroendocrine tumor (NET), stage 1, diagnosed by gastric biopsy and immunohistochemistry. 

Evans Syndrome is an autoimmune condition which attacks erythrocytes and thrombocytes. It is most frequently found as an idiopathic presentation. It may also be evident simultaneously during the course of other diseases [6], such as a paraneoplastic syndrome of aggressive tumors. 

First and foremost, at a first glance, it is clear that there is a positive response of the platelet count to the administration of octreotide. Octreotide is a somatostatin analogue which has been widely used for the treatment of NETs and upper gastrointestinal hemorrhage. It acts directly upon the somatostatin receptors and inhibits its hormonal secretion [7]. It is considered a first-line treatment for the control of symptoms in carcinoid tumors [8]. In this case, it may be possible that octreotide itself is responsible for the temporary remission of the idiopathic thrombocytopenia purpura (ITP). It may be behaving with some type of immunomodulatory effect or lymphocyte-regulating mechanism. To date, this property remains unknown, yet it may be a starting point for a potential area of further study and investigation. 

Leaving aside the hypothetical assumptions of octreotide, further analysis regarding the gastric NET and the Evans Syndrome must be made. The Evans Syndrome could be interpreted as a paraneoplastic manifestation of the gastric NET. Paraneoplastic syndromes (PNS) are characterized by anteceding the first manifestations of the proper neoplastic disease, in some cases, years before the diagnosis of the neoplastic process itself [9]. Nonetheless, the scientific literature does not describe Evans Syndrome as a typical paraneoplastic manifestation of neuroendocrine carcinomas. This association has been found in isolated and very rare cases [10]. The most common PNS associated with NETs are those caused by the overproduction of hormones, such as Cushing Syndrome, hypercalcemia, hypersecretion of cytokines and cerebellar degeneration [5, 11]. Furthermore, most PNS are dependent on the original neoplasm itself—something that is not seen in this case because the scan for remnant NET gave a negative result. 

Surely, the patient has a strong autoimmune background in her organism, endorsed by her diagnosis of hemolytic anemia and ITP. Considering this autoimmune background, and independent from its origin, it is possible to suspect, and only suspect, pernicious anemia (PA). PA of autoimmune origin generates a chronic atrophic gastritis [12, 13], which leads to the hyperplasia of enterochromaffin and G cells, finally predisposing the gastric tissue to the generation of metaplasia and abnormal tumoral cells [14]. Association between gastric NETs and PA has been described [15–17], yet little has been noted about the coexistence of PA and ITP [18, 19]. In this case, this patient did not have a specific workup for PA, so such diagnosis cannot be made. Nonetheless, such PA workup should have been executed based upon the patient's intrinsic susceptibility for autoimmunity and a gastric biopsy with atrophic gastritis. 

## Figures and Tables

**Table 1:** Test results for initial and differential diagnoses.

Laboratory exam	Result	Reference values
ANA	Negative	>INM 02
ANCA		
MPO	Negative	>INM 08
PR3	Negative	>INM 08
Polyspecific direct coombs	Positive (3+)	—
Monospecific direct coombs		
IgG	Positive (3+)	—
IgM	Negative	—
IgA	Negative	—
C3d	Positive (2+)	—
C3c	Negative	—
Chromogranin A	142.0 ng/mL	1.9–15.0
Rheumatoid factor	Negative	>INM 05
Gastrin	927.0 ng/L	0–100
VIH 1-2 Ab + Ag p24	Not reactive	—
Haptoglobin	99 mg/dL	5–220
Full hepatitis panel (HBV/HCV)	Not reactive	—
CMV	IgG negative	—
	IgM negative	—
HSV 1 and 2	IgG negative	—
	IgM negative	—
VDRL	Not reactive	>INM 10
Bone marrow aspiration/biopsy	Hyperplastic bone marrow with signs of peripheral thrombocytosis	

## List of Abbreviations

NET: Neuroendocrine tumor
5-HIAA: 5-Hydroxyindoleacetic acid
SQ: Subcutaneous
CBC: Complete blood count
Tc99: Technetium-99
ITP: Idiopathic thrombocytopenic purpura
PA: Pernicious anemia
ANA: Antinuclear antibody
ANCA: Antineutrophil cytoplasmic antibody
MPO: Myeloperoxidase
PR3: Proteinase 3.
